# An Assessment of the Impact of Land Thermal Infrared Observation on Regional Weather Forecasts Using Two Different Data Assimilation Approaches

**DOI:** 10.3390/rs10040625

**Published:** 2018-04-18

**Authors:** Li Fang, Xiwu Zhan, Christopher R. Hain, Jifu Yin, Jicheng Liu, Mitchell A. Schull

**Affiliations:** 1 (National Oceanic and Atmospheric Administration) NOAA/ (National Environmental Satellite, Data, and Information Service) NESDIS/ (Center for Satellite Applications and Research) STAR, 5830 University Research Court, College Park, MD 20740, USA; 2 Earth System Science Interdisciplinary Center, University of Maryland, 5825 University Research Court, College Park, MD 20740, USA; 3 NASA Marshall Space Flight Center, Redstone Arsenal, Huntsville, AL 35812, USA

**Keywords:** land surface temperature, data assimilation, ensemble Kalman filter (EnKF), ALEXI

## Abstract

Recent studies have shown the unique value of satellite-observed land surface thermal infrared (TIR) information (e.g., skin temperature) and the feasibility of assimilating land surface temperature (LST) into land surface models (LSMs) to improve the simulation of land-atmosphere water and energy exchanges. In this study, two different types of LST assimilation techniques are implemented and the benefits from the techniques are compared. One of the techniques is to directly assimilate LST using ensemble Kalman filter (EnKF) data assimilation (DA) utilities. The other is to use the Atmosphere-Land Exchange Inversion model (ALEXI) as an “observation operator” that converts LST retrievals into the soil moisture (SM) proxy based on the ratio of actual to potential evapotranspiration (fPET), which is then assimilated into an LSM. While most current studies have shown some success in both directly the assimilating LST and assimilating ALEXI SM proxy into offline LSMs, the potential impact of the assimilation of TIR information through coupled numerical weather prediction (NWP) models is unclear. In this study, a semi-coupled Land Information System (LIS) and Weather Research and Forecast (WRF) system is employed to assess the impact of the two different techniques for assimilating the TIR observations from NOAA GOES satellites on WRF model forecasts. The NASA LIS, equipped with a variety of LSMs and advanced data assimilation tools (e.g., the ensemble Kalman Filter (EnKF)), takes atmospheric forcing data from the WRF model run, generates updated initial land surface conditions with the assimilation of either LST- or TIR-based SM and returns them to WRF for initializing the forecasts. The WRF forecasts using the daily updated initializations with the TIR data assimilation are evaluated against ground weather observations and re-analysis products. It is found that WRF forecasts with the LST-based SM assimilation have better agreement with the ground weather observations than those with the direct LST assimilation or without the land TIR data assimilation.

## 1. Introduction

Land surface temperature (LST, or “skin temperature”), as a key parameter of the Earth’s surface energy balance, is one of critical variables in weather and climate models [1–3]. LST influences the land-atmosphere interaction by controlling upward terrestrial radiation and affecting the surface latent and sensible heat flux exchanges through which it affects the planetary boundary layer and atmospheric convection. Accurate LST information is therefore of great value to potentially improving the surface-atmosphere water and energy balance and upper level temperature and humidity, and all in turn to improve the accuracy of weather and climate forecasts. Real-time satellite products are capable of providing spatially-continuous observations of surface parameters while accurately capturing the dynamics of surface conditions. Satellite retrievals of LST are widely available from thermal infrared (TIR) sensors onboard on polar-orbiting and geostationary platforms. Several satellite LST products are operationally available for use in the numerical weather prediction (NWP) models, such as Geostationary Operational Environmental Satellite system (GOES) LST [4], Meteosat Second Generation (MSG) LST [5–6] and GOES Surface and Insolation Products (GSIP) LST [7]. In this research, TIR observations from the GOES series are collected to be assimilated into the weather forecast model.

Current studies have shown some success in directly assimilating LST into LSMs [8–11]. Attempts have been made to assimilate LST by adjusting model terms [10, 12–13] For instance, Bosilovich (2007) assimilated LST into the Common Land Model (CLM) by introducing an incremental bias correction term into the model’s surface energy budget [10]. This method uses the increments to adjust the model background temperature, turbulent fluxes and terrestrial long-wave radiation. It is found that the LST assimilation improved 2-m air temperature estimates, both in mean and variability. McNider (1994) adjusted terms in the surface energy balance of atmospheric models in response to satellite LST retrievals based on a regional-scale atmospheric model. Another major path to assimilating LST uses an adjoint model [9, 14–15]. Meng (2009) used the land surface energy balance as the adjoint model to adjust the evaporative fraction of the first soil layer and canopy according to in situ LST observations from four AmeriFlux sites [9]. Improvement in ET is found in the verification results. This type of assimilation method is, however, not easy to implement, especially in complex land surface models (e.g., Noah LSM). Reichle et al. (2010) conducted satellite LST assimilation in offline Noah and Catchment LSMs using the EnKF method [8]. Modest, yet statistically-significant improvements of up to 0.7 K in RMSE and 0.05 K in the anomaly R were found with the assimilation of dynamic bias-corrected satellite retrievals of LST. Later on, Draper et al. (2014) demonstrated LST assimilation into the Catchment LSM using a dynamic approach to addressing observation-minus-forecast mean differences [16]. Skin temperature is diagnosed from the energy balance equation in the Noah LSM, which is presented very differently from the Catchment model where the LST is a prognostic variable. It makes the application of EnKF data assimilation of satellite LST in the Noah LSM more complicated. Few studies have targeted the direct assimilation of LST into the Noah LSM in recent literature.

Even as certain success has been shown in directly assimilating LST into LSMs in current studies, significant issues have been found [8–9, 16]. One challenge comes from the absolute biases between satellite-based LST and modeled surface temperature due to the inconsistencies of satellite LST retrieval algorithms and model physics and parameterizations used to predict LST within LSMs. These biases need careful consideration for the direct assimilation of satellite-based LST to avoid unrealistic estimation of sensible and latent heat fluxes. An additional challenge is that surface skin temperature exhibits a very short memory as a model state variable. If the biases and inconsistencies of LST observations are not fully addressed (between model and observation), the model state will quickly return to its pre-assimilation state, which will ultimately limit the impact of a direct LST assimilation.

Therefore, there are compelling reasons to test alternative approaches to assimilating pertinent information of LST into operational NWP models. A number of studies has successfully developed methodologies to exploit LST for monitoring soil moisture (SM) conditions [12–13, 17–22]. It has been found that an LST-based SM signal is particularly advantageous because it is able to provide SM information over moderate to dense vegetation, a capability that is limited with microwave sensors [23]. Anderson et al. (2007) and Hain et al. (2009; 2011) have outlined a technique for simulating the effects of SM on latent heat estimates from the Atmosphere-Land Exchange Inversion model (ALEXI), which is forced with an observation of the morning change in LST, using a stress function relating the value of the fraction of actual to potential evapotranspiration (fPET) to soil moisture [17, 19, 23]. The ALEXI model is built on the two-source energy (TSEB) approach of Norman et al. (1995), which partitions the composite surface radiometric temperature into characteristic soil and canopy temperatures, based on the fraction of vegetation cover [24–27]. In ALEXI, the lower boundary conditions for the two-source model are provided by TIR observations taken at two times during the morning hours from a geostationary platform such as GOES.

Anderson et al. (2007; 2011) demonstrated that spatial distributions in the fraction of actual to potential ET correlate well with patterns in precipitation-based indices, responding to rainfall events at monthly time steps [17, 28]. The ALEXI fPET product has been utilized as a thermal infrared (TIR)-based SM proxy signal for assimilation using an EnKF in the Noah LSM [18]. Importantly, the assimilation of TIR-based SM retrievals from ALEXI is able to avoid some of the issues that can hamper the direct assimilation of LST. For instance, the biases in absolute values of satellite-based LST retrievals are effectively circumvented by ALEXI because it is sensitive only to the time rate of change of mid-morning LST [24]. The assimilation of ALEXI SM retrievals also circumvents the difficulties in comparing satellite-based and model-based LST, which is an inevitable and complicated step in approaches for direct assimilation of satellite-based LST. Finally, soil moisture has been shown to be a more stable state variable for data assimilation than LST.

Notably, as most of the existing studies (either direct assimilation of LST- or TIR-based SM assimilation) focus on the offline land surface models, a direct application of LST data products through data assimilation in NWP operations has not been well demonstrated. The assimilation of satellite-based observations through the coupled NWP-LSM modeling system could potentially lead to significant forecast improvements. This study is therefore focusing on the assessment of the benefits from direct LST assimilation and LST-based SM assimilation on weather forecast. The primary objectives of this study are: (1) to assess the impact on satellite-based land surface thermal infrared observations on regional numerical weather predictions and (2) to explore the differences between direct assimilation of LST and LST assimilation using the ALEXI model as an observation operator to convert LST information to soil moisture.

With the tools of the NASA Land Information System (LIS) [29–30] and the NASA-Unified Weather Research and Forecasting (NU-WRF) modeling system [31], a semi-coupled LIS/WRF system is developed to assimilate LST and/or LST-derived SM in LIS semi-coupled with WRF. A series of data assimilation experiments is performed: (1) an open-loop run with no data assimilation; (2) a data assimilation (DA) run with direct assimilation of GOES LST; and (3) another DA run with the assimilation of SM derived from LST using the ALEXI model. Section 2 introduces the semi-coupled data assimilation system in detail. Section 3 describes the satellite, meteorological forcing and validation datasets used in this study. The data assimilation experiments design is presented in Section 4. Data assimilation results and their validation are given in Section 5. Conclusions and discussion are given at the end of the paper.

## 2. Semi-Coupled LIS and WRF Data Assimilation System

### 2.1. Land Information System

The NASA Land Information System (LIS) is a flexible software framework to integrate satellite and ground-based observations and advanced LSMs to accurately characterize land surface states and fluxes [29–30]. The land surface modeling infrastructure in LIS consists of several well-documented LSMs (Noah, CLM, Catchment, Mosaic et al.), which typically run in an uncoupled model using a combination of observationally-based precipitation, radiation, meteorological and land surface parameter datasets.

Additionally, LIS employs advanced data assimilations tools such as the ensemble Kalman filter (EnKF). The EnKF provides a flexible approach for incorporating errors in the model and observations, and its ensemble-based treatment of errors makes it suitable for handling the modestly non-linear dynamics and the temporal discontinuities that are typical of land-surface processes. It is widely accepted as an effective technique in recent research for sequential assimilation of hydrologic variables such as soil moisture, skin temperature, snow cover, etc. Therefore, the EnKF method is adopted in this study to conduct the land data assimilation.

The details of assimilating LST and soil moisture within LIS will be given in Sections 4.2 and 4.3, respectively. Noah LSM [32] will serve as the core land surface model in data assimilation experiments in this study because it is currently implemented in the operational land surface model for the numerical weather prediction at the National Center for Environmental Prediction (NCEP) [32–33]. Specifically, the Noah model Version 3.3 will be employed for land data (LST and SM) assimilation within LIS using the EnKF technique, and it will also serve as the core land component in the standard NCAR Advanced Research WRF (WRF-ARW) model for forecasting.

### 2.2. NASA Unified Weather and Research Forecast Model

The NASA-Unified WRF (NU-WRF) modeling system developed at NASA Goddard Space Flight Center (GSFC) is an observation-driven integrated modeling system representing aerosol, cloud, precipitation and land processes at satellite-resolved scales [31]. The NU-WRF (Version 7) adopted in this study incorporates the standard NCAR Advanced Research WRF (ARW) Version 3.5.1 and LIS (v7.0rp1) into a unified framework with distinct advantages of: (1) setting up long-term spin up land surface conditions on the common grid as the WRF forecast domain; (2) providing LIS land simulations with near-surface forcing from the parent WRF run; (3) easy replacement of updated initial conditions from LIS output to WRF.

### 2.3. LIS Semi-Coupled with WRF

**Figure 1.**
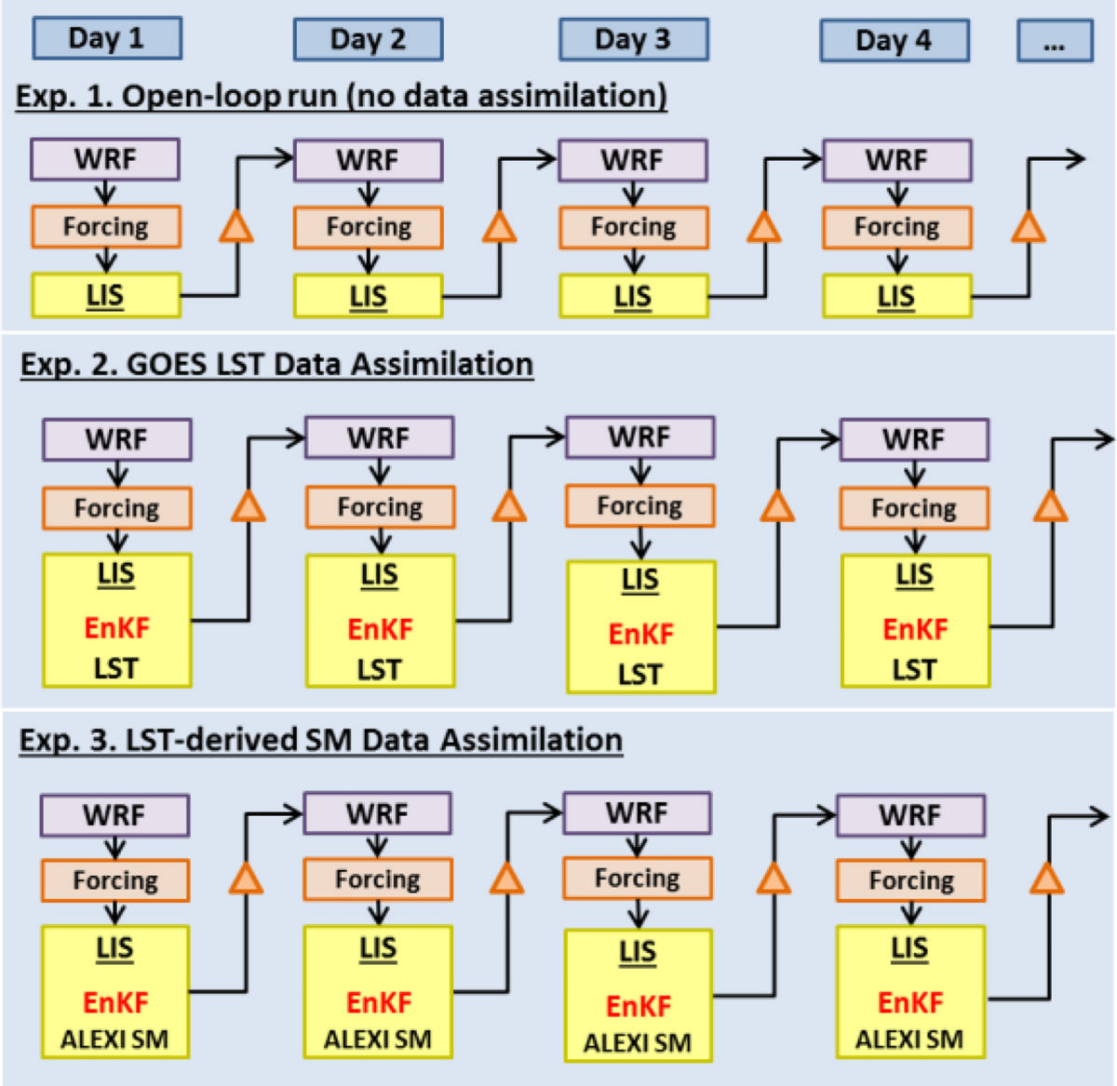
Schematic description of the semi-coupled Land Information System (LIS) and Weather Research and Forecast (WRF) system for the three data assimilation experiments. SM, soil moisture.

With the tools, a semi-coupled LIS/WRF framework is designed to test the impact of either direct LST or LST-based SM assimilation on WRF weather forecast. In the coupling workflow, WRF provides atmospheric forcing data to LIS, and LIS sets up the simulation domain on the same grid (spatial resolution and projection) with the same terrestrial data and land surface physics (identical versions of the Noah LSM) as the WRF run. All land data assimilations (e.g., LST DA and SM DA) are conducted within LIS using the EnKF method. LIS then generates updated initializations daily and returns updated initial land surface data (e.g., SM, soil temperature, fluxes, albedo, etc.) to WRF for next day forecasts. Figure 1 shows a schematic of the semi-coupled LIS/WRF system. Three assimilation experiments will be conducted: (1) open-loop run, which uses no data assimilation; (2) LST DA, which directly assimilates LST retrievals; and (3) SM DA, which assimilate LST-based ALEXI SM retrievals. Following the above-mentioned workflow, initializations of land states are updated with the assimilation of either GOES LST or ALEXI SM, but with near-surface forcing from the parent WRF run. The daily updated land states will impact the subsequent forecasts in response to the changes in soil moisture and temperature. More details of direct LST assimilation and LST-based SM assimilation within the semi-coupled system will be presented in Sections 4.2 and 4.3, respectively.

## 3. Datasets

### 3.1. LST-Derived Soil Moisture

The Atmosphere-Land Exchange Inverse (ALEXI) model is used as an “observation operator” to convert remotely-sensed thermal infrared signals into the land surface SM condition. The ALEXI model exploits the mid-morning rise in LST to deduce the land surface energy balance by partitioning the composite surface radiometric temperature into characteristic soil and canopy temperatures, based on the fraction of vegetation cover [24–25, 27].

Anderson et al. (2007) and Hain et al. (2009; 2011) outlined a technique for simulating the effects of SM on latent heat estimates from ALEXI through an SM stress function based on the derived estimate of the fraction (fPET) between actual to potential evapotranspiration (ET) [17, 19, 23]. Actual ET is estimated from the ALEXI model, and potential ET is estimated using a Penman–Monteith formulation based on the observed temperature, wind speed and net radiation at the surface [17, 24]. A simple evaporative stress index (ESI), which represents anomalies in the ratio of actual-to-potential ET (fPET), can then be developed from ALEXI flux estimates. ESI has a value of zero when there is ample moisture/no stress and a value of one when evapotranspiration has been cut off because of stress-induced stomatal closure and/or complete drying of the soil surface. The anomalies in fPET have been demonstrated to be well correlated with a suite of standard precipitation-based drought indicators [28]. The fPET product is used as the ALEXI SM proxy for the inter-comparison and validation in this study.

Maps of ALEXI SM retrievals were generated at 8-km resolution over the North America domain using LST and shortwave/longwave radiation from the GOES-R proxy products. Fang et al. [34] have conducted a comprehensive validation by comparing the ALEXI SM data with three microwave (MW)-based satellite SM products (merged, active and passive MW products from ESA’s Climate Change Initiative (CCI)) and Noah model simulations. It is found that SM retrievals of the ALEXI model based on TIR satellite observations demonstrate skills equivalent to all the MW satellite retrievals and slightly better over certain regions with moderate to high vegetation cover where microwave signals suffer significant attenuation issues over vegetation. Supported by the National Environmental Satellite, Data, and Information Service (NESDIS) Satellite Product and Services Review Board, the GOES ET and Drought Product System (GET-D) is operational at the Office of Satellite and Product Operations (OSPO) at NOAA, to generate daily ET, the ALEXI-based SM proxy data and drought maps since September 2016.

### 3.2. GOES Land Surface Temperature

There are many land surface thermal infrared observations from satellite sensors (e.g., the imagers or sounders on NOAA GOES satellites, MODIS on NASA Terra and Aqua satellites). In this study, the hourly thermal infrared observations from the GOES imager are used for LST DA tests to keep the same thermal data source as those used in ALEXI SM derivations. Observations from GOES Imager are available at the Comprehensive Large Array-data Stewardship System (CLASS) of NOAA. Specifically, LST information is derived from GOES observations at 11 micron channel through atmospheric correction using meteorological data from the NCEP Climate Forecast System (CFS). The same atmospheric correction algorithm is used for derivation of hourly LST for the direct LST DA experiment and the two morning LSTs used in the ALEXI model in the ALEXI SM DA test. This setting avoids the potential LST inconsistency impacts on DA effects. Strict quality control and cloud contamination removal techniques have been used in the LST derivation approach to filter out low-quality and cloudy pixels. The LST retrieval algorithm is applied to GOES East and West observations separately. In the ALEXI model, GOES East and West LSTs are merged together to obtain the full coverage of the study domain. As for the overlap area where observations from both sensors are available, the observation from the satellite with a smaller viewing angle was chosen. In direct LST DA experiments, GOES East LST will be assimilated into WRF at the 45th minute at each hour after being converted to soil temperature, while observations from the GOES West satellite will be assimilated at every sharp hour (00th).

### 3.3. Ground Weather Observational Data for WFR Forecast Validation

The performance of assimilating land thermal infrared observations is assessed using ground weather observations including near surface variables and precipitations. Specifically, the Global Upper Air and Surface Weather Observations from the National Center for Environmental Prediction (NCEP) are collected for the evaluation of surface and near surface temperature and humidity forecasts. These observations are composed of a global set of surface and upper air reports operationally processed by the NCEP, including pressure, geopotential height, temperature, dew point temperature, wind direction and speed with the time intervals ranging from hourly to 12 hourly. The data are archived in the Global Upper Air and Surface Weather Observations (PREPBUFR) format available at the National Center for Atmospheric Research, Computational and Information Systems Laboratory (Boulder, Colorado, CO, USA). http://rda.ucar.edu/datasets/ds337.0. Additionally, NCEP National Stage IV Precipitation Analysis [35–36] is used to evaluate precipitation forecasts from each of the WRF runs. Stage IV is a mosaic of regional multi-sensor analysis product produced by NWS River Forecast Centers. The real-time, 4-km, hourly/six-hourly Stage IV product is archived at the National Center for Atmospheric Research, and further details of the product can be found at http://www.emc.ncep.noaa.gov/mmb/ylin/pcpanl/.

## 4. Data Assimilation Experiments

To assess the impact of assimilating either land surface temperature observations or LST-based soil moisture on regional numerical weather prediction, a set of data assimilation experiments is carried out based on the LIS and WRF semi-coupling framework, including: (1) an open-loop run with no data assimilation, (2) an LST DA run that directly assimilates LST observations and (3) an SM DA run that assimilate SM derived from LST using the ALEXI model. In all of these experiments, WRF provides atmospheric forcing data to LIS; LIS runs the land surface process simulation and data assimilation using the same version of the Noah LSM as WRF; and then, LIS returns the updated land state variables and fluxes back to WRF to initialize its forecasts.

### 4.1. Open-Loop Run of the LIS-WRF System

The open-loop run, which uses no data assimilation, could serve as a good benchmark for assessment of the land TIR data assimilations and the effect of land-atmosphere coupling. The study domain is configured at 12-km spatial resolution in Gaussian projection over North America. The LIS-WRF runs are conducted for 15 days from 1–15 June, 2012. From 8 June, WRF forecasts for each time step (four steps a day for seven days) are used to evaluate the impacts of the data assimilation. This period is of particular interest because of the severe drought that occurred across the whole Continental United States (CONUS). Satellite observations of LST are not available when cloud cover exists. For those locations or days when LST data are missing, either of the two LST DA methods is not applied. If no LST DA is carried out for all locations all the time, the WRF forecasts for the open-loop and DA runs should be the same.

Each model run is set up for 48-h forecasts, with hourly outputs starting from a fixed initialization time (0600 UTC). The three-hourly North American Regional Reanalysis (NARR) data are employed as forcing data to initialize the ARW model runs. The model configuration is summarized in detail in Table 1. The settings of the model grid, physical process schemes and boundary conditions in the LST DA and ALEXI SM DA runs are kept the same as those in open-loop except for surface initialization updates by land surface temperature assimilation.

### 4.2. LST Data Assimilation

The direct assimilation of GOES LST information requires testing of the DA utility to address a couple of science issues before a credible impact assessment can be obtained. Firstly, skin temperature in the Noah model is represented as a diagnostic variable from the surface energy balance equation with no associated heat capacity. Therefore, an additional connection between the diagnostic skin temperature and a prognostic variable in the model is required to implement the data assimilation increments so that final model forecasts can be altered by the assimilation. Reichle et al. (2010) tried to apply increments to the prognostic soil temperature at the top layer with a thickness of 10 cm [8]. The issues regarding the replacement of top layer soil temperature with skin temperature include: (1) misrepresenting soil temperature at a 10-cm depth as the skin temperature at a very thin layer of the land surface; and (2) the phase shift between soil temperature and LST.

**Figure 2.**
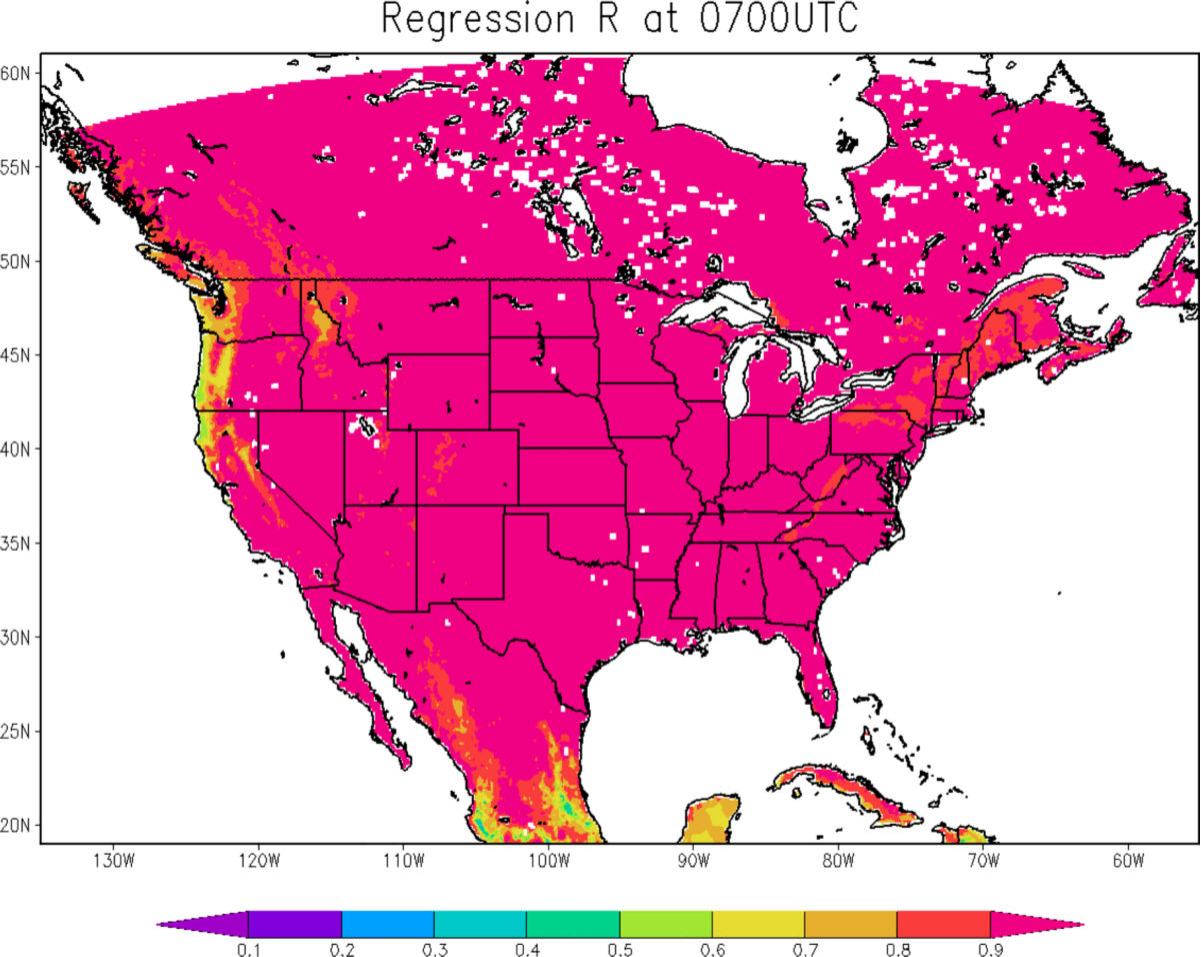
Regression R^2^ between skin temperature and top layer soil temperature simulations from the Noah LSM, at 0700 UTC.

The strategy of direct LST assimilation in our study was inherited from Reichle’s method using the prognostic variable of top layer soil temperature as a connector. However, certain improvements are made to deal with the misrepresenting issue in Reichle’s approach. Instead of direct use of skin temperature as the top layer soil temperature, a function is built to convert skin temperature to soil temperature to alleviate the mismatching and phase shift issues. The regression is firstly built from the Noah LSM simulations between top layer soil temperature and skin temperature over 12-year climatology from 2001–2012. The relationship is then applied to project satellite LST retrievals to soil temperature (top layer) prior to the assimilation. The diurnal cycles of the surface temperature and soil temperature must be preserved, and therefore, the relationships are built independently for each hour of the day. The map of regression R^2^ at 0700 UTC is presented in Figure 2, which illustrates the high representativeness of the regression over almost the entire domain with coefficients larger than 0.9.

The EnKF approach has been demonstrated to be well suited to the non-linear and intermittent character of land surface processes [37–38] and broadly used in land data assimilation [18, 39–45]. In our application, the EnKF module in the NASA Land Information System (LIS; [30]) is utilized with a 20-member ensemble to assimilate LST information (both direct LST assimilation and TIR SM assimilation) into the Noah model.

### 4.3. LST-Based ALEXI SM Data Assimilation

ALEXI-based SM retrievals and LSM SM estimates can exhibit large differences in climatological statistics. The success of data assimilation methods depends on unbiased satellite observations relative to the model state predictions, and thus, proper treatment of systematic errors (bias) is critical for the success of data assimilation. The system biases can be alleviated by employing a bias correction technique that rescales a given ALEXI SM retrieval to a value that is statistically consistent with the distribution of the Noah LSM SM dataset. There are several methods handling the biases prior to data assimilation. The standard normal deviate-based scaling method is used in our study, which requires the computation of the first two statistical moments (mean and variance) for each dataset. A normalized anomaly is calculated for the dataset and rescaled to volumetric soil moisture content consistent with the identical normalized anomaly in the LSM SM climatology based on the Noah mean and standard deviation. For a given ALEXI retrieval product, this linear transformation is represented by:

(1) $$\theta_{\mathrm{ALEXI}/\mathrm{Noah}}=\mu_{\mathrm{Noah}}+\left(\theta_{\mathrm{ALEXI}}-\mu_{\mathrm{ALEXI}}\right)\frac{\sigma_{\mathrm{Noah}}}{\sigma_{\mathrm{ALEXI}}}$$

where θ_ALEXI/Noah_ is the re-scaled ALEXI SM retrieval, θ_ALEXI_ is the raw soil moisture retrieval, μNoah and μALEXI are the mean of Noah and the ALEXI retrievals, respectively, and σNoah and σALEXI are the standard deviation of Noah and the ALEXI retrievals.

## 5. Results

### 5.1. Differences in WRF Forecasts with and without Assimilations

**Figure 3.**
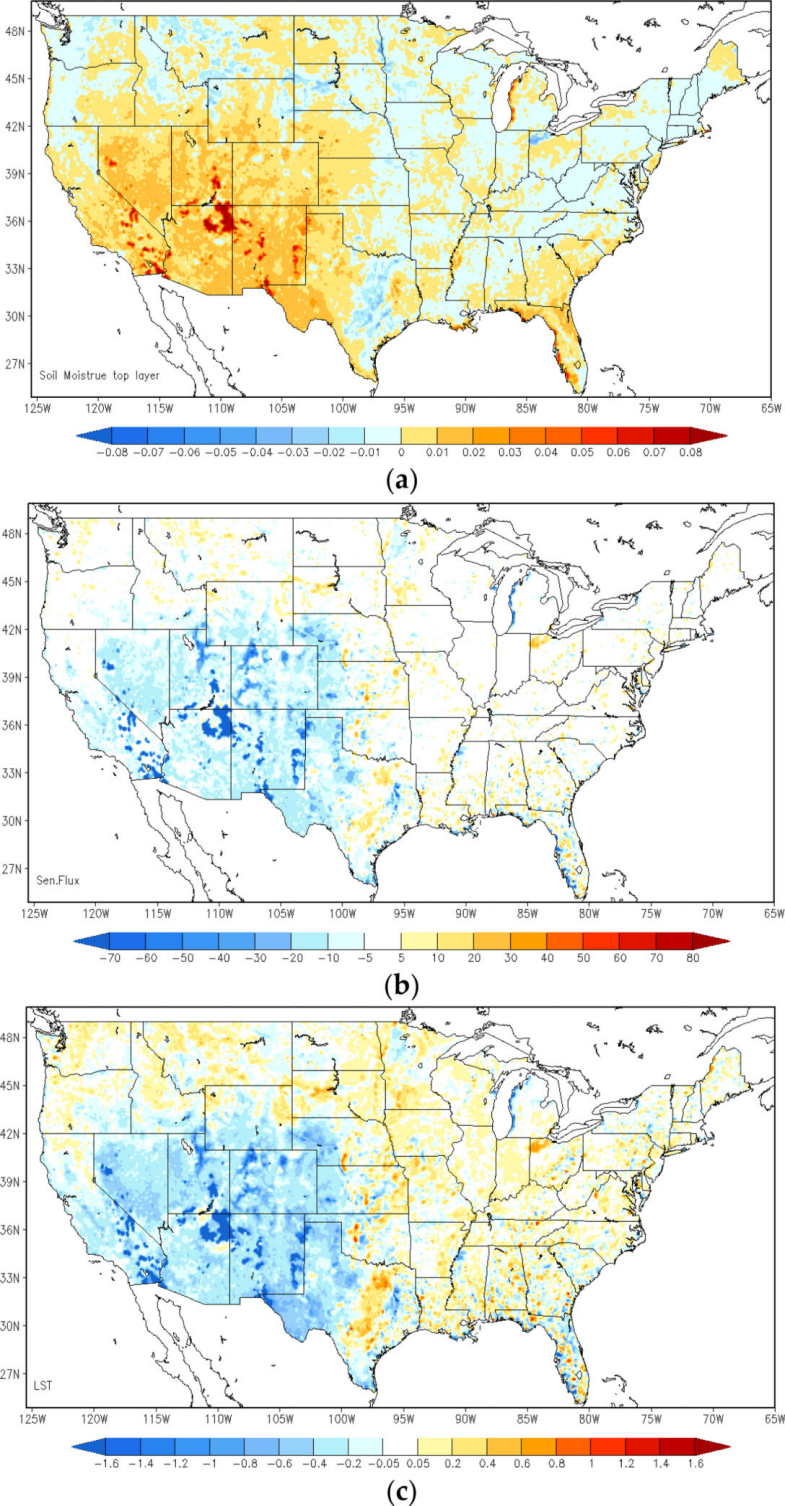

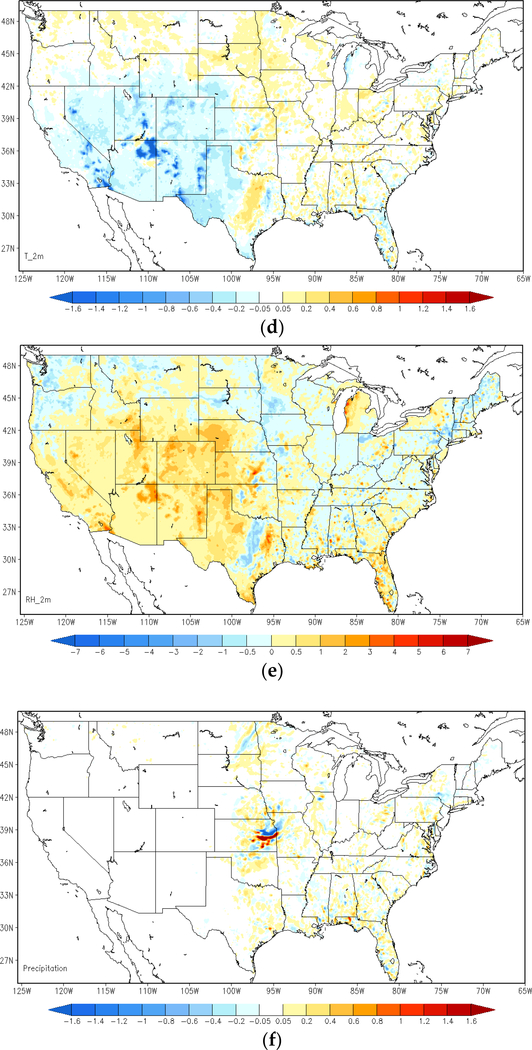
Comparison between the LST-based ALEXI SM data assimilation (DA) run and the open-loop run; average difference of top layer soil moisture (**a**), sensible heat flux (b**)**,; surface skin temperature (**c**), 2-m air temperature (T-2m) (**d**), 2-m relative humidity (RH-2m) (**e**) and precipitation (**f**) at 1900 UTC, over the period of 8–15 June 2012 (ALEXI SM DA run minus open-loop run).

The assimilation of LST observations into the weather forecast model can substantially adjust the model simulations. Figure 3 shows the differences in estimates of top-layer soil moisture, sensible heat flux, forecasts of land surface temperature, 2-m temperature (T-2m), 2-m relative humidity (RH-2m) and precipitation between the ALEXI SM DA run and the open-loop run. The differences are averaged from forecasts at 1900 UTC over the period from 8–15 June 2012. The WRF model forecasts are altered significantly over most of the study domain by the assimilation of ALEXI SM information with soil moisture becoming wetter over the western U.S. and slightly dryer over the central and eastern areas. The impact on 2-m temperature presents a similar pattern as that on skin temperature, but of a smaller magnitude. The largest impact can be detected over the southwest after ALEXI SM assimilation, particularly in the Southern Mountain Region and Southern West Coast, where surface temperature forecast drops on the order of 2 K–3 K and relative humidity rises around 3%. Moderate changes can be found over the central and eastern U.S. with increased forecasts of surface temperature and a gentle drop of humidity.

**Figure 4.**
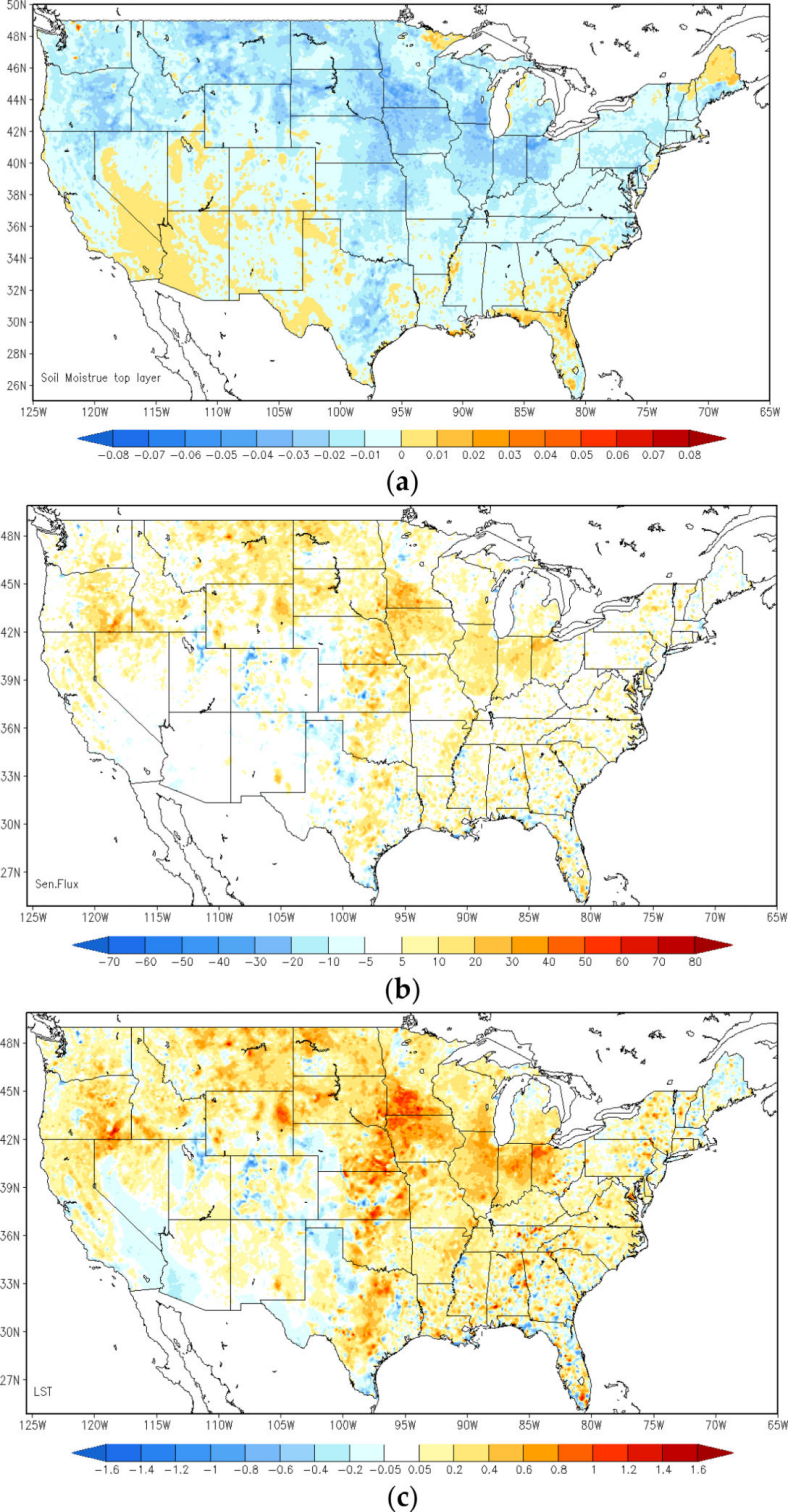

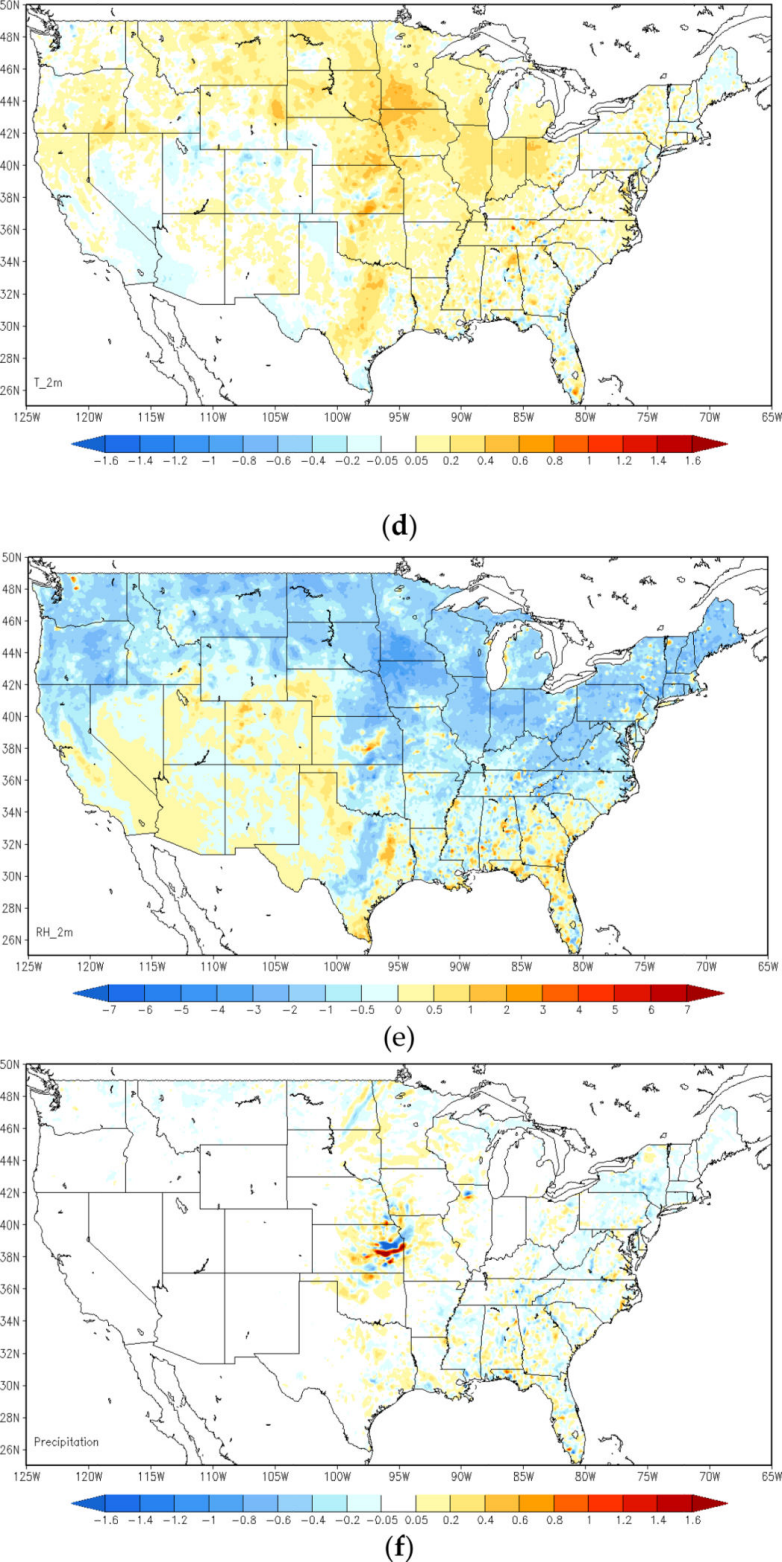
Comparison between the LST DA run and the open-loop run; average difference of top layer soil moisture (**a**), sensible heat flux (**b**), surface skin temperature (**c**), T-2m (**d**), RH-2m (**e**) and precipitation (**f**) at 1900 UTC, over the period of 8–15 June 2012 (LST DA run minus open-loop run).

Figure 4 is the same as Figure 3, but for the differences between the direct LST data assimilation run and the open-loop run. A substantial rise in surface and 2-m temperature has seen over the Northern Plains and the Midwest regions, while 2-m relative humidity has fallen by as much as 5% correspondingly over those regions. Additionally, temperature predictions slightly cool down in small regions at the border among California, Nevada and Arizona, as well as the central area in Texas. The direct LST assimilation presents higher surface temperature forecasts over the Southern Mountain and Plains regions, which is similar to the impacts from ALEXI SM DA, but of a much smaller magnitude.

### 5.2. Evaluation of the WRF Forecasts against Ground Observations

#### 5.2.1. Two-Meter Air Temperature

**Figure 5.**
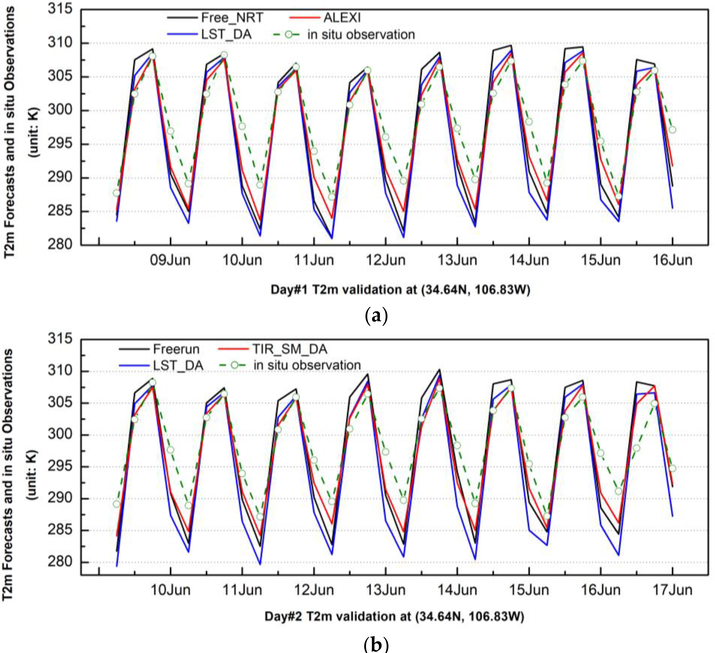
Comparison of 2-m temperature between forecasts and in situ observations over the validation site in New Mexico (34.64N, 106.83W) for Day 1 forecast (**a**) and Day 2 forecast (**b**).

To assess the performance of assimilation runs, model forecasts are validated using in situ observations. Figure 5 shows the comparison of time series of 2-m temperature forecasts from the ALEXI SM DA run and the LST DA run along with the in situ observation at a sample validation site (34.64N, 106.83W) in New Mexico over the period of 8–16 June 2012. The 2-m temperature forecast of the WRF open-loop run has an apparent warm bias over daytime and a cold bias over nighttime during the validation period. The bias reaches as high as 2.28 degrees at daytime and 4.8 degree at nighttime on average. Evidently, the assimilation of satellite LST information, either direct LST assimilation or LST-based SM assimilation, corrects the warm bias. The warm bias is reduced by 1.5 K on average with direct assimilation of LST, while the assimilation of LST-based SM data can further correct the warm bias by 0.5 K on top of the direct LST assimilation. Furthermore, the assimilation of ALEXI SM is also beneficial to 2-m temperature forecasts by correcting cold bias during nighttime significantly. It is, however, found that the direct LST assimilation degraded the nighttime forecasts by dragging the 2-m temperature even lower than the already cold bias simulations. Although the biases shown in both day’s forecasts have a similar pattern (warm bias at daytime and cold bias at night time), it is notable that the bias on the Day 2 forecast is generally larger than that on Day 1 by 0.53 degrees on average. It is reasonable and understandable that the errors can be accumulated along time in the WRF predictions.

**Figure 6.**
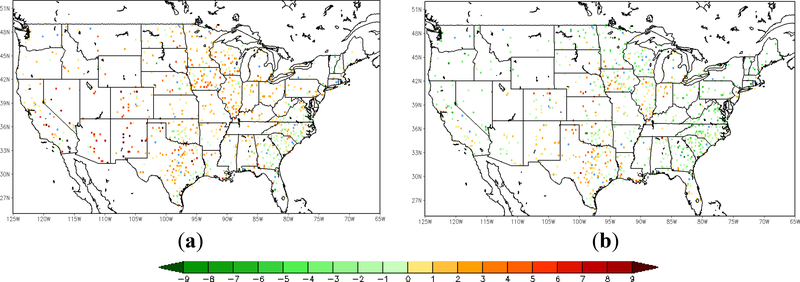
The difference in the RMSE of T2m forecasts between the WRF open-loop run and DA runs; open-loop run minus TIR SM DA run (a); open-loop run minus LST DA run (b); warm (cool) color means added (degraded) value; sites marked blue did not exhibit a statistically-significant difference (43 stations for TIR SM DA and open-loop; 41 stations for LST DA and open-loop; 986 stations in total).

**Figure 7.**
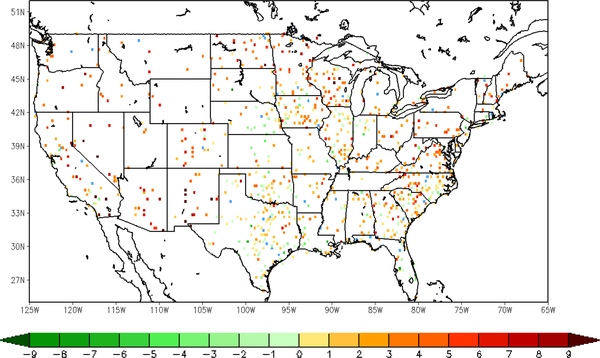
Difference in the RMSE of T2m forecasts from the LST DA run and the TIR SM DA run (LST DA minus TIR SM DA); warm (cool) color means added (degraded) value; sites marked blue did not exhibit a statistically-significant difference (47 stations out of 986).

Following the comparison of the sample site shown above, an overview of validation results over all the in situ sites is presented here, using nearly a thousand ground observations collected from the Global Upper Air and Surface Weather Observations. The spatial distribution of the relative differences in root-mean-square-error (RMSE) of 2-m temperature forecast between the LST-based SM DA (direct LST DA) run and the WRF open-loop run is shown on the left (right) in Figure 6. The differences between each of the three datasets are tested for statistical significance at a confidence interval of 95%. The RMSE difference is calculated by the WRF open-loop run minus DA runs, with positive (negative) values meaning that forecasts the from open-loop run show larger (smaller) error than those from DA runs. The sites with warm (cool) color shown in the map are where the data assimilation presents added (degraded) value in the 2-m temperature forecasts. Sites that did not exhibit a statistically-significant difference are marked blue. There are about 45 sites (out of 985 in total) with no significant differences according to *t*-test results with a significance level of 0.05. Around 70.26% validation sites show a positive impact with the assimilation of LST-based ALEXI SM data. Moderate improvement can be found in Arizona, New Mexico and Texas, and yet, degradation is shown at a small amount of sites over the southeastern coast, such as Georgia and South Carolina. In general, it is promising to notice that the majority of validation sites over the central and western U.S. show slight improvement. The direct assimilation of LST, on the other hand, does not present an overall positive impact on 2-m temperature forecasts as shown in Figure 6 on the right. Except for the sites in Texas and part of Iowa, direct assimilation of LST degraded the WRF forecasts at most of the sites over both the eastern and western areas. TIR-based SM DA performs better over 78.74% of sites compared to the direct LST DA run (Figure 7). The error statistics shown here are the daily average based on 6-h interval in situ observations. Even though the direct LST assimilation presents a positive impact during the daytime, e.g., correcting daytime warm bias in the case shown above, the degradation during nighttime cancelled out the overall performance.

#### 5.2.2. Two-Meter Relative Humidity

**Figure 8.**
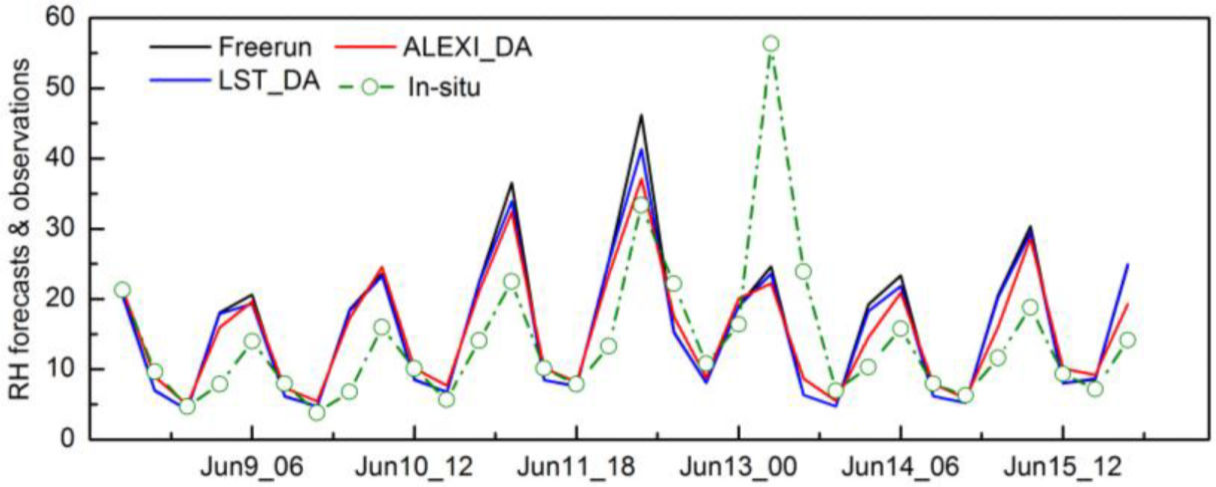
Comparison of the 2-m temperature between forecasts and in situ observations at a sample site at the validation site in New Mexico (34.64N, 106.83W) for the Day 1 forecast.

The potential impact of assimilating LST in the NWP model is then assessed by validating the near surface forecast of atmospheric humidity. Figure 8 exhibits the comparison of relative humidity (RH) forecasts from the WRF open-loop run and two assimilation runs, along with the observations from the ground validation site in New Mexico (106.83W, 34.64N) over the period of 8–12 June 2012.

The open-loop run presents a consistently positive bias compared to the in situ measurements. There is a dramatic jump to as high as almost 60% in relative humidity on 13 June in the in situ record, but it is not shown in any model runs. The region in New Mexico and Texas was under a severe drought during that period, which makes the extremely high value questionable. It may be a noisy signal caused by unknown observational reasons, so the data on 13 June are excluded in the following analysis. Except for that day, the time-series comparison suggests that the assimilation of LST information (both direct LST and LST-based SM) is able to reduce the positive bias. Compared to the mean bias from the open-loop run (3.07%), the direct LST assimilation can reduce bias to 2.62%, while the LST-based ALEXI SM assimilation run lowers the bias to 2.53%. The largest impact occurred on 12 June, when the bias of the open-loop run is reduced by 4.85% with directly assimilating LST and an additional 9.10% reduction by assimilating LST information into the WRF model by converting to SM signals (ALEXI SM).

**Figure 9.**
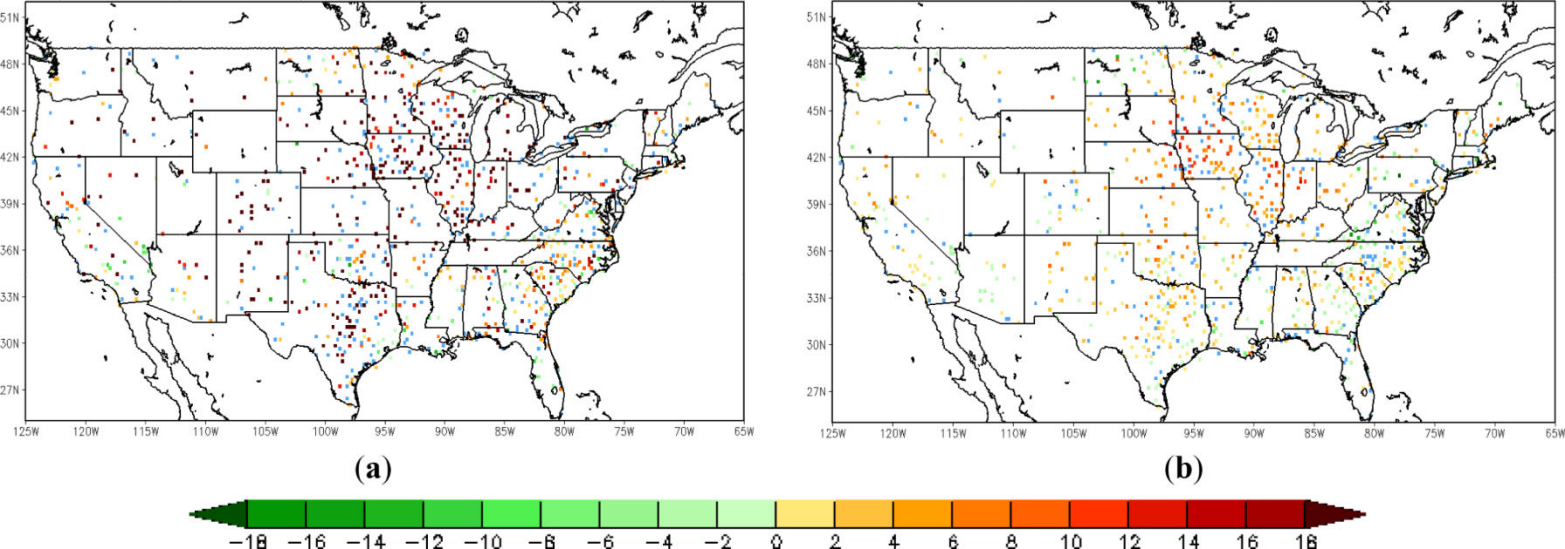
Difference in the RMSE of 2-m relative humidity forecasts between the WRF open-loop run and DA runs; open-loop run minus TIR SM DA run (a); open-loop run minus LST DA run (b); warm (cool) color means added (degraded) value; sites marked blue did not exhibit a statistically-significant difference (246 stations for TIR SM DA and open-loop; 192 stations for LST DA and open-loop; 970 stations in total)

**Figure 10.**
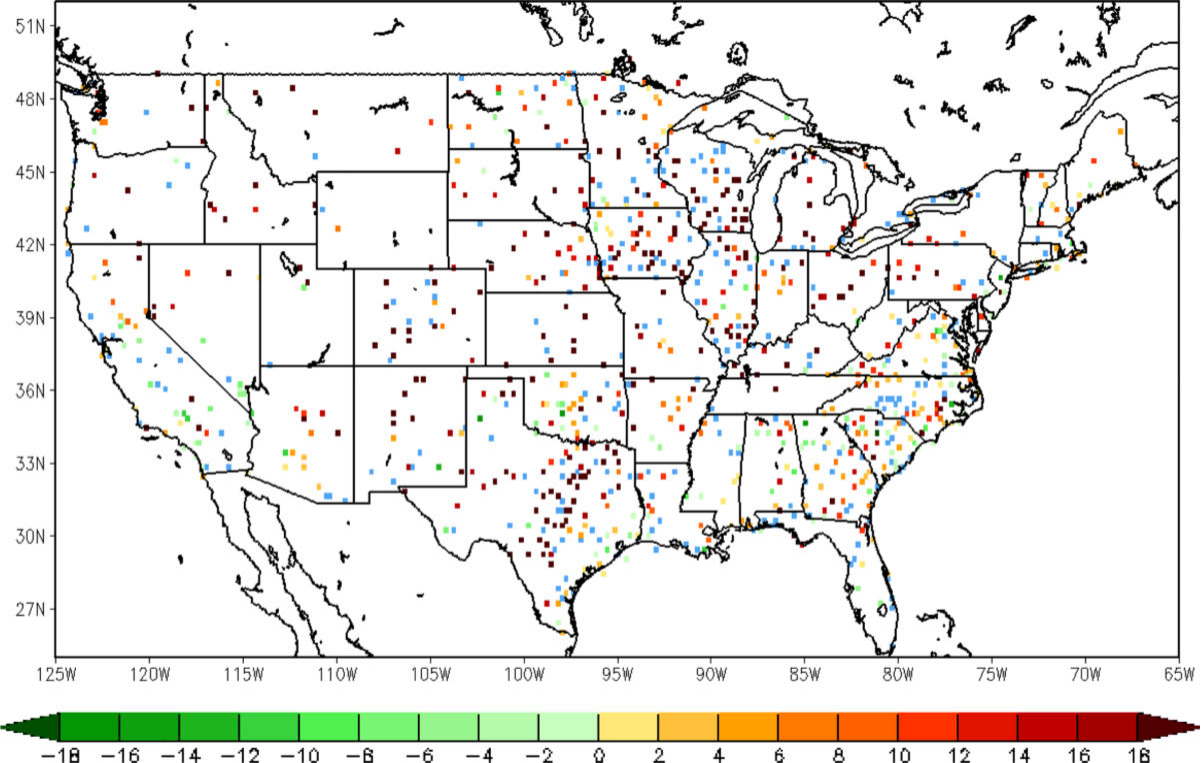
Difference in the RMSE of 2-m relative humidity forecasts from the LST DA run and the TIR SM DA run (LST DA minus TIR SM DA); warm (cool) color means added (degraded) value; sites marked blue did not exhibit a statistically-significant difference (248 stations out of 970).

Similarly to the analysis of the near surface temperature, the spatial distribution of the difference in RMSE of the 2-m RH forecasts is given here with more than 900 validation sites over the CONUS domain in Figure 9. The RMSE difference between the LST-based SM DA (direct LST DA) run and the WRF open-loop run is shown on the left (right) in Figure 9. The inter-comparison between assimilations of LST-based SM and direct LST is shown in Figure 10. The differences of over around 200 ground sites out of a total of 970 are not statistically significant at the 95% confidence interval, which are marked in blue color in Figures 9 and 10. The positive impact with the assimilation of land surface temperature is gained over the majority of the validation sites, while slight degradation can be detected in a small amount of sites along the eastern coast area. Furthermore, the magnitude of improvement with LST-based SM DA is larger than that with direct LST DA by roughly 1.70% on average.

#### 5.2.3. Daily Precipitation

**Figure 11.**
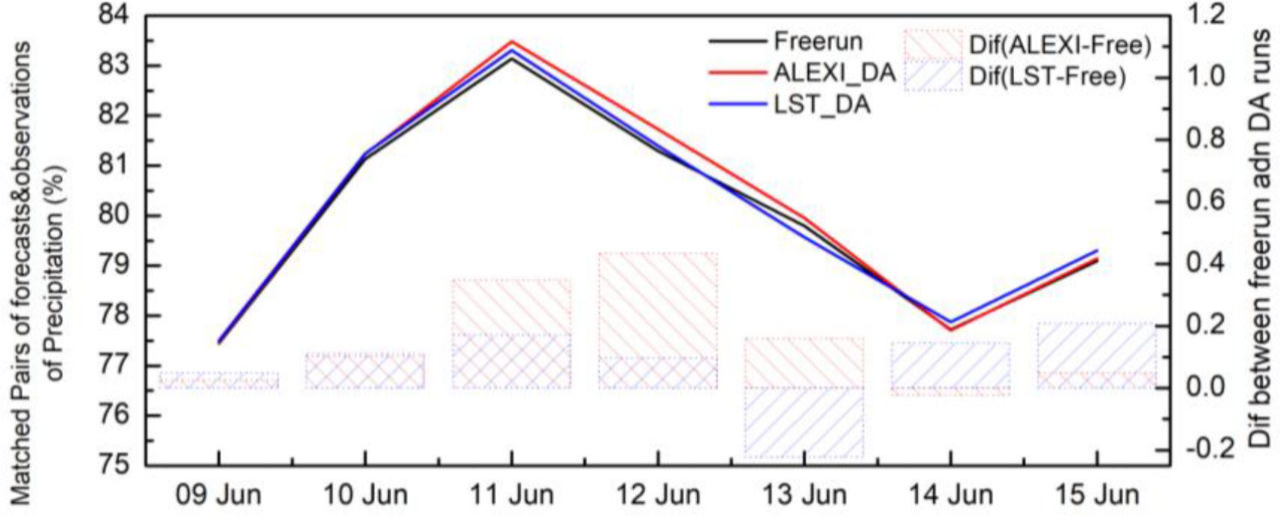
Percentage of matched pairs of the Stage IV precipitation dataset and WRF forecasts (open-loop run, LST DA run and direct LST DA run).

Lastly, the impact of the assimilation of LST information on precipitation is examined by comparing WRF runs with the NCEP National Stage IV Precipitation dataset. The percentage of matched pairs of 24-h accumulated precipitation between the Stage IV precipitation dataset and WRF forecasts is shown in Figure 11. First of all, the impact on precipitation by assimilation of land surface temperature is not as strong as the above-analyzed near surface forecasts (e.g., temperature or humidity). However, the validation results still illustrate subtle improvement in precipitation forecast from LST assimilation runs by showing a relatively higher percentage of matched pairs of forecasts and ground observations, averaged over the CONUS domain. Better agreement with ground observations can be detected throughout the validation period, except one day on 13 June when direct LST assimilation shows slight degradation.

**Figure 12.**
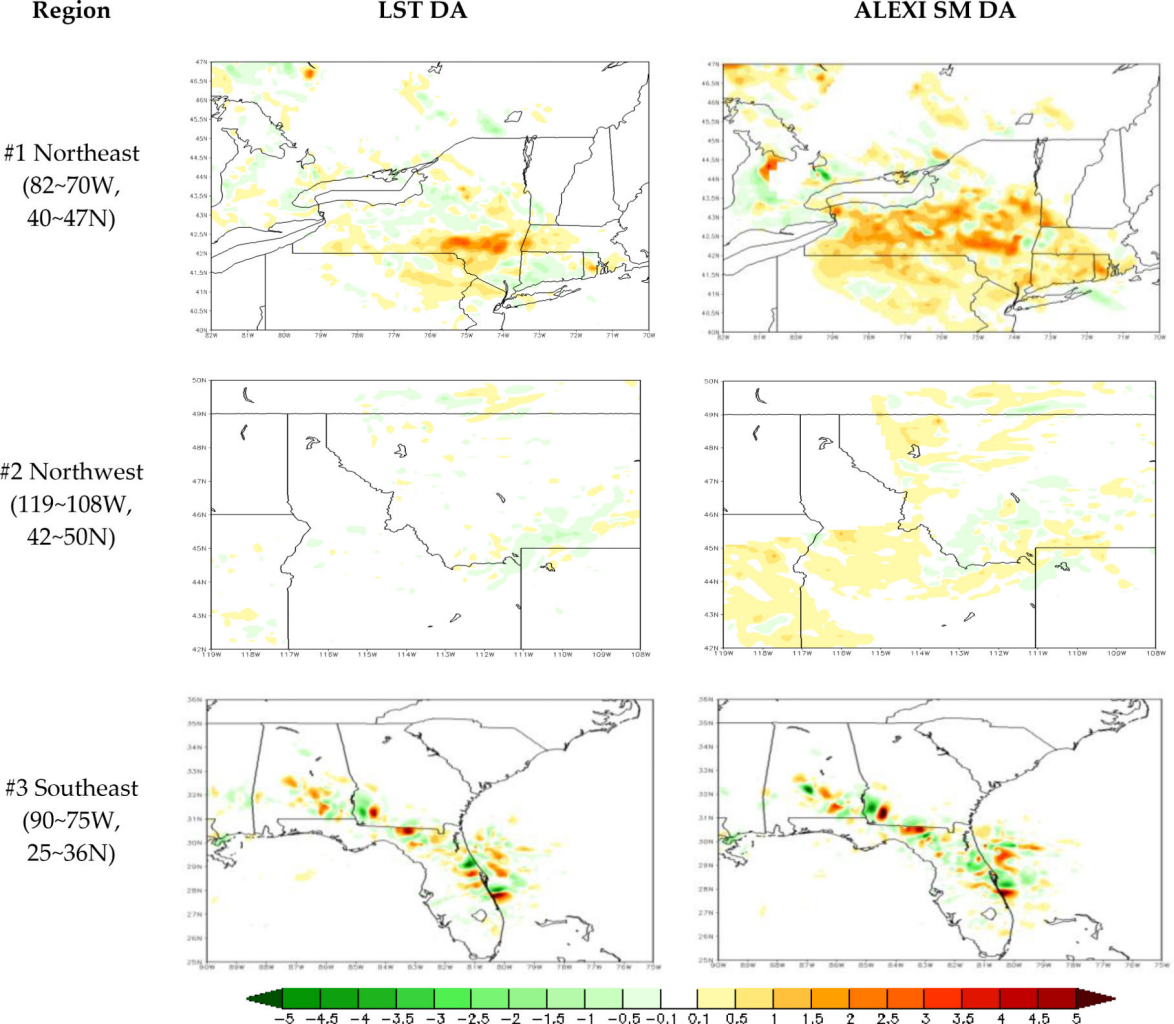
RMSE difference of precipitation forecasts between the WRF open-loop run and the direct LST DA run (LST-based SM DA run) on the left (right) panel for three major rainfall events across CONUS; warm (cool) color means added (degraded) value.

The accuracy of model forecasts (with and without DA) is further quantitatively analyzed by comparing them with the National Stage IV Precipitation dataset pixel by pixel. We focus on three major rainfall events across CONUS on 10 June 2012 in this analysis. The RMSE is calculated for each of the WRF runs (open-loop run and two DA runs) separately using Stage IV precipitation as the “true” observations. The RMSE differences between DA runs and the open-loop run (open-loop run minus DA runs) are then plotted and shown in Figure 12 to illustrate the added or degraded value by data assimilations. The RMSE differences between direct LST DA (LST_based SM DA) and the open-loop run are shown on the left (right) panel in Figure 12, with warm (cool) color meaning improvement (degradation). The maps of RMSE differences demonstrate that both assimilation approaches improve the precipitation forecasts by showing less RMSE over the majority of the rainfall region, compared to the open-loop run, especially over Region #1 and #2. Evidently, LST-based SM DA outperforms the direct LST approach, presenting added value to precipitation forecasts over larger areas and to a larger extent over the northeast (Region #1) and northwest regions (Region #2). Two types of LST assimilation are comparable over rainfall events in the southeast region (Region #3), with mixed added and degraded values after assimilation.

#### 5.2.4. Validation Summary

The overall performances of the two assimilation approaches (direct LST DA and LST-based SM DA) are summarized in Table 2 in terms of mean RMSE differences and the number of sites with improvement, compared to the open-loop run. The accuracy of three forecast variables (T2m, RH-2m and precipitation) are examined using in situ observations, and the average RMSE differences between DA runs and the open-loop run are computed at both regional and CONUS scales.

As for the T-2m forecast, the RMSE of the ALEXI-based SM DA run drops by 1.58% compared to the errors of the open-loop run, averaged from all validation sites combined on the CONUS domain. Over 70% of validation sites (nearly 1000 sites in total) show improvement with the assimilation of ALEXI-based SM. The direct assimilation of LST, on the other hand, has seen slight degradation on the T-2m forecast with an increase in RMSE by 0.97% over the CONUS domain. Both assimilation approaches present added value to RH-2m forecasts, decreasing RMSE by 1.82% with the thermal-based SM assimilation method and by 1.68% with the direct LST assimilation. The magnitude of impact is substantially higher over the TX-NW region, an area of particular interest because it is affected by a significant drought over that period. The introduction of real-time satellite LST/SM information is expected to have a greater impact on that particular region. The validation results from the thermal-based SM assimilation are encouraging, reducing the RMSE of T-2m (RH-2m) by as much as 2.65% (0.68%), with around 72.29% (80.45%) of sites showing improvements. The outperformance of the direct LST assimilation method is found for over 60% of validation sites for the T-2m forecast and nearly 75% for RH-2m, compared to the open-loop run. The assimilation of satellite LST information did not significantly improve precipitation forecasts, and yet, a relatively higher hit rate and skill score are achieved from DA runs compared to the open-loop run.

## 6. Discussion and Summary

Accurate forecasts of numerical weather prediction models rely on the quality of the initialization of land surface state variables (e.g., soil moisture, soil temperature, and surface temperature). However, current weather forecast models that are operational at NOAA/NCEP have not yet assimilated satellite-based land variables’ observations. A semi-coupled LIS/WRF system is proposed in this study to examine the impact of satellite retrievals of LST on weather forecasts. In this semi-coupled system, WRF provides atmospheric forcing data to LIS, and LIS is responsible for land data assimilation (LST/SM) using the EnKF technique; WRF in turn receives the land surface states (SM, green vegetation fraction, fluxes, albedo, etc.) updated in LIS. Considering the known challenges of satellite-based LST assimilation caused by the unique characteristics of satellite LST information, this research evaluates and compares two approaches of assimilating satellite retrievals of LST. One is to directly assimilate LST retrievals into models using EnKF. The other unique methodology is to convert LST information into the soil moisture proxy using the ALEXI model before assimilation.

Sensitivity analysis is carried out firstly. Difference fields of near surface temperature and humidity demonstrate that the assimilation of land thermal information led to substantial changes in surface energy exchanges, lower-level thermal condition, surface soil moisture and soil temperature. The variations in the updated land surface initialization after data assimilation affect the evolution in the NWP model and alter the prediction of near surface simulations and ultimately precipitation.

Following sensitivity analysis, WRF forecasts with and without LST information assimilations are validated and compared using in situ observations from nearly a thousand ground sites. Validation on 2-m air temperature forecasts shows that the assimilation of satellite LST information, either direct LST assimilation or LST-based SM assimilation, corrects the daytime warm bias to a great extent. Slight improvement is gained at the majority of validation sites over the central and western U.S. with the assimilation of LST-based SM. Even though the direct LST assimilation shows a certain positive impact during the daytime, obvious degradation can be found in the nighttime. The validation on 2-m relative humidity exhibits similar conclusions, and an overall positive impact is found. The impact of the assimilation of LST information on precipitation is lastly assessed by comparing with NCEP National Stage IV Precipitation Analysis. The impact of LST assimilation on precipitation is fairly subtle, compared to that on near surface simulations. It is, however, concluded that the location and intensity of precipitation from assimilation runs are more closely aligned with the Stage IV analysis, compared to those from the open-loop run.

This study attempts to test the effectiveness of using the ALEXI model as an observation operator to assimilate satellite land surface temperature observations in numerical weather prediction models with a relatively short study time period. A more comprehensive investigation with longer time periods or other different climatological events should be further carried out before the tested LST data assimilation methods can be applied to operations. Additionally, our study found that using the ALEXI model as an observation operator in the LST DA does change the effectiveness of the LST DA, although the improvements resulting from the DA using ALEXI as the observation operator may not be statistically significant for the time period of this study. It is expected that more a comprehensive investigation will be stimulated from this study on LST data assimilation. Future studies shall also address: (1) the design of the full coupled land data assimilation utility within WRF; (2) issues in direct LST assimilation with the Noah LSM, where LST is not presented as a prognostic variable.

In summary, validation results indicated that: (1) the assimilation of satellite LST information (either direct LST assimilation or LST-based SM assimilation) corrects the daytime warm bias of T-2m forecasts; (2) the validation on 2-m relative humidity exhibits similar conclusions, and an overall positive impact is found; (3) the impact of LST assimilation on precipitation is fairly subtle, compared to that on near surface simulations; and (4) ALEXI-based SM assimilation, which avoids the issues caused by the direct assimilation of LST information, presents larger improvements in the surface field simulations (surface temperatures and specific humidity) and precipitation forecasts.

## Figures and Tables

**Table 1.** LIS-WRF model configuration details. ARW, Advanced Research WRF.

Variables	Assignment
WRF dynamical core	Advanced Research WRF
grid spacing/projection	12 km/Lambert
dimension (west-east by south-north)	480 × 400
Integration time step	24 s (the same in LIS and ARW)
Vertical dimension	42
number of soil levels or layers	4
Land usage	MODIS (20-category)
Microphysics	5 (Eta microphysics: the operational microphysics in NCEP models)
Land surface	Noah (v3.3)
Planetary boundary layer	Mellor-Yamada-Janjic scheme

**Table 2.** Summary of the performances of the two assimilation approaches.

Forecasts	Approaches	Difference in Normalized RMSE* Compared to Open-Loop Run (Percentage)		Number of Sites with Improvements Compared to Open-Loop Run (Percentage)	
		TX-NM Region	CONUS	TX-NM Region	CONUS

T-2m	TIR SM DA	2.65	1.58	79.58	72.29
	LST DA	0.68	−0.97	61.26	36.09

RH-2m	TIR SM DA	16.22	13.10	82.72	80.45
	LST DA	1.82	1.68	74.87	69.07

**Forecasts**	Approaches	Difference in Hit rate** Compared to Open-Loop Run		Difference In RMSE Compared To Open-Loop Run	

Precipitation	TIR SM DA	0.16		0.18	
	LST DA	0.08		0.04	

* Difference in normalized RMSE = (RMSE__openloop_ − RMSE__DA_)/RMSE__openloop_, with a positive (negative) value meaning that DA cases show improvements (degradation)

** Difference in hit rate = (H__DA_ − H__openloop_)/RMSE__openloop_, with a positive (negative) value meaning DA cases show improvements (degradation)
